# The Side Effects of Therapeutic Radioiodine-131 on the Structure of Enamel and Dentin in Permanent Human Teeth

**DOI:** 10.3390/biology10040284

**Published:** 2021-04-01

**Authors:** Alexandru Mester, Marioara Moldovan, Marian Taulescu, Codruta Sarosi, Ioan Petean, Adriana Vulpoi, Andra Piciu, Andrada Voina-Tonea, Cristina Moisescu-Goia, Elena Barbus, Doina Piciu

**Affiliations:** 1 Department of Oral Health, University of Medicine and Pharmacy “Iuliu Hatieganu”, 400012 Cluj-Napoca, Romania; mester.alexandru@umfcluj.ro; 2 Department of Polymer Composites, Institute of Chemistry “Raluca Ripan”, University Babes-Bolyai, 400294 Cluj-Napoca, Romania; marioara.moldovan@ubbcluj.ro (M.M.); liana.sarosi@ubbcluj.ro (C.S.); 3 Department of Pathology, University of Agricultural Sciences and Veterinary Medicine, 400372 Cluj-Napoca, Romania; marian.taulescu@usamvcluj.ro; 4 Department of Chemistry and Chemical Engineering, University Babes-Bolyai, 400294 Cluj-Napoca, Romania; ioan.petean@ubbcluj.ro; 5 Nanostructured Materials and Bio-Nano-Interfaces Center, Interdisciplinary Research, Institute of Bio-Nano-Sciences, University Babes-Bolyai, 400271 Cluj-Napoca, Romania; adriana.vulpoi@ubbcluj.ro; 6 Department of Medical Oncology, University of Medicine and Pharmacy “Iuliu Hatieganu”, 400012 Cluj-Napoca, Romania; 7 Department of Dental Materials, University of Medicine and Pharmacy “Iuliu Hatieganu”, 400012 Cluj-Napoca, Romania; 8 Department of Endocrine Tumors and Nuclear Medicine, Oncology Institute “Ion Chiricuta”, University of Medicine and Pharmacy “Iuliu Hatieganu, 400012 Cluj-Napoca, Romania; mg.cristina@iocn.ro (C.M.-G.); elena.barbus@umfcluj.ro (E.B.); doina.piciu@umfcluj.ro (D.P.)

**Keywords:** radioiodine-131, I-131, dental structure

## Abstract

**Simple Summary:**

Our research suggest radioiodine-131 used for differentiated thyroid carcinomas has an impact on the oral health of patients. We found that alteration of dental hard tissues starts after 6 h post-radioiodine administration. These findings highlight the importance of the multidisciplinary team management in the quality of life of the oncological patient.

**Abstract:**

Radioiodine-131 (I-131) is an essential therapy for patients with differentiated thyroid carcinomas (DTC). Generally, I-131 is safe and well tolerated, but patients may present early or late complications in the oral and maxillofacial areas. Thus, the aim of this study was to evaluate in-vitro, the alteration of enamel and dentin after I-131 exposure using histopathological assessment, scanning electron microscopy (SEM) and atomic force microscopy (AFM). For I-131 irradiation, an in-vitro protocol was used that simulates the procedure for irradiation therapy performed for patients with DTCs. A total of 42 teeth were divided into seven groups (n = 6) and irradiated as follows: control, irradiation groups (3, 6, 12, 36, 48 h, 8 days). Histological changes were observed at 48 h (enamel surface with multifocal and irregular areas) and at 8 days (enamel surface with multiple, very deep, delimited cavities). SEM imaging revealed the enamel destruction progresses along with the treatment time increasing. The alterations are extended into the enamel depth and the dislocated hydroxyapatite debris is overwhelming. The enamel-dentine interface shows small gaps after 6 h and a very well developed valley after 12 h; the interface microstructure resulted after 8 days is deeply altered. The AFM imaging shows that I-131 affects the protein bond between hydroxyapatite nano-crystals causing loss of cohesion, which leads to significant increasing of nano-particles diameter after 6 h. In conclusion, both enamel and dentin appear to be altered between 12 and 48 h and after 8 days of treatment are extended in depth.

## 1. Introduction

Radioiodine-131 (I-131) therapy has been used for decades in treating hyperthyroidism, Grave’s disease and well differentiated thyroid malign pathologies [1,2,3]; due to increasing incidence of the thyroid cancer, the use of I-131 is significantly higher in the last years [4]. Although radioiodine is widely used for its therapeutic effects and in general well accepted by the human organism, several late side effects have been described over the years. These side effects can occur because of the external action of the radiation or can be linked to the immune system of the patient or to the function of the thyroid gland [4].

In the oral and maxillofacial field side effects of radioiodine therapy represent a challenge, not only because of their nature, but also because of the unpredictable response of irradiated organisms and slow healing rates [5]. Alterations of the salivary glands function have been reported after radioactive iodine treatment. It was proven that salivary glands have the capacity to retain iodide and pass it into the salivary flow. Of all the salivary glands, the parotid gland is the most vulnerable to radiation [6] and will exhibit an accentuated form of sialadenitis, which represents the most common complication of radioiodine therapy [7]. Salivary duct stenosis can also play an important role in the appearance of obstructive sialadenitis symptoms [8]. For this reason, one of the constant met repercussion is xerostomia [9], manifested trough dry mouth complains of the irradiated patients. The occurrence and the precedency of Sjogren Syndrome should be therefore evaluated, before initiation of radioactive iodine therapy [10]. All these manifestations are often accompanied by deterioration of the taste capacity, trough hypogeusia or dysgeusia [11].

Even though healthcare providers embrace and apply protective attitudes towards these specific manifestations, chronic evolutions of the pathologies were observed, affecting the long-lasting health status and wellbeing [12]. Based on the insufficient salivary flow, patients who undertake radioiodine therapy can also develop oral mucositis, microbial or fungal colonization of the mucosa, periodontal pathologies or carious lesions [13] or dental fractures [14].

It was previously proven that radiation of head and neck malign pathologies can lead to dentin denudation, due to enamel exposure. The deterioration of the hard dental structures, with the alteration of the interprismatic tissue, is dependent of the radiation dose [15]. Also a modification can be observed, regarding the proportion of proteins and minerals in the structure of enamel and dentin [15]. However, studies regarding the explicit influence of radioiodine therapy on the teeth structure are not many. Older animal research have showed that I-131 is able to infiltrate the enamel, dentin, pulp tissue and periodontal structure [16], while, newer studies on human dental structure demonstrated that the radiation activity rises exponentially from 3 to 24 h after irradiation, with the modification of the hydroxyapatite crystals after 12 h [16]. Nonetheless, the topic of radioiodine therapy influence on human hard dental tissue can be further expanded. In regards to this statement, the aim of this study was to assess, in-vitro, the alteration of enamel and dentin after I-131 exposure using histopathological assessment, scanning electron microscopy (SEM) and atomic force microscopy (AFM).

## 2. Materials and Methods

### 2.1. Specimen Preparation

After approval of the ethical institutional board (contract number 17/12.02.2020), all patients included were aware of our research protocol and signed the informed consent regarding the dental extraction and using their data and samples in scientific purposes. The included teeth had the following characteristics: maxillary incisors without decays/fillings, endodontic treatment, or prosthetic crown. After dental extraction, all specimens were washed with water, if necessary, dental calculus was removed with ultrasonic scaling, immersed in ultrasonic bath (for 5 min), then dried and examined on microscope (10× magnification); at the end of this protocol, specimens were immersed in artificial saliva (4 °C 1 month). The specimens were randomly assigned in groups (each group was composed of 6 teeth): control group and irradiation groups up 192 h.

### 2.2. Radioiodine Irradiation Protocol

For radioiodine-131 irradiation, we used an in-vitro protocol that simulates the procedure for radioiodine therapy performed for patients with differentiated thyroid carcinomas (DTC) [17]. The amount of radioiodine activity was calculated to be similar to that administered in the ablation therapies of DTC. The estimated absorbed dose in the remnant tissue is 300 Gy and the dose in the blood stream is considered to be 2 Gy [18]. The teeth are submitted to a similar dose of 2–2.8 Gy as the blood stream [19]. The protocol consisted in a solution of 16 mCi (592 MBq) I-131 dissolved in 50 mL of artificial saliva. In the prepared solution, the specimens were introduced and then, after irradiation, were taken out at 3, 6, 12, 36, 48 h and 8 days. The control specimens were emerged in 30 mL of artificial saliva without I-131.

### 2.3. Histological Assessment of Dental Tissues

At the end of each day of experiment, the teeth were taken out from the artificial saliva (control group) and from the I-131 solution (experimental groups), washed with water, dried and then stored in 10% phosphate-buffered formalin, for 48 h. After this process, within each group, 3 specimens were taken out; the roots were embedded in self-cured acrylic resin templates, up to 2 mm below cementoenamel junction, in order to simulate the alveolar bone. When the resin was ready, each specimen was taken out from the template and each crown was sectioned longitudinally in the mesio-distal direction resulting in two parts (buccal, lingual) using a diamond saw cooled with water mounted in a precision saw (Isomet, Buehler, Lake Bluff, IL, USA) resulting in sections of 1 mm width. For histological examination, an Axio Scope A1 microscope (Zeiss, Oberkochen, Germany) was used. The photomicrographs were taken using an Axiocam 208 color digital camera and ZEN core imaging software from Zeiss.

### 2.4. Scanning Electron Microscopy and Atomic Force Microscopy Analysis of Dental Tissues

For SEM and energy dispersive X-ray spectroscopy (EDX) investigation, the samples previously sectioned with an IsoMet 1000 (Buehler) microtome at 1 mm, were covered with gold, for a better visualization. SEM and chemical analysis of local area by EDX were carried out with a FEI Quanta 3D FEG (FEI Company, Hillsboro, OR, USA) dual beam microscope. AFM was used to investigate the nano-structural changes within the enamel and dentine at several treatment times. The investigation was performed on a JSPM 4210 Scanning Probe Microscope (Jeol, Tokyo, Japan), using the intermittent contact—tapping mode. The used cantilevers are NSC 15 type MikroMasch (Sofia, Bulgaria), and exhibit a resonant frequency of 325 kHz and a force constant of about 40 N/m. The topographic images were recorded at a scanned area of 2.5 × 2.5 μm to assure an optimal view of the structures at the nano level. The scanning rate vary from 1 to 1.5 Hz. The images were processed in the standard manner using Jeol Win SPM (version 2.0). The roughness (Ra and Rq) and nano-particles diameter were measured with the soft for each image.

## 3. Results

### 3.1. Histological Findings

**Figure 1:**
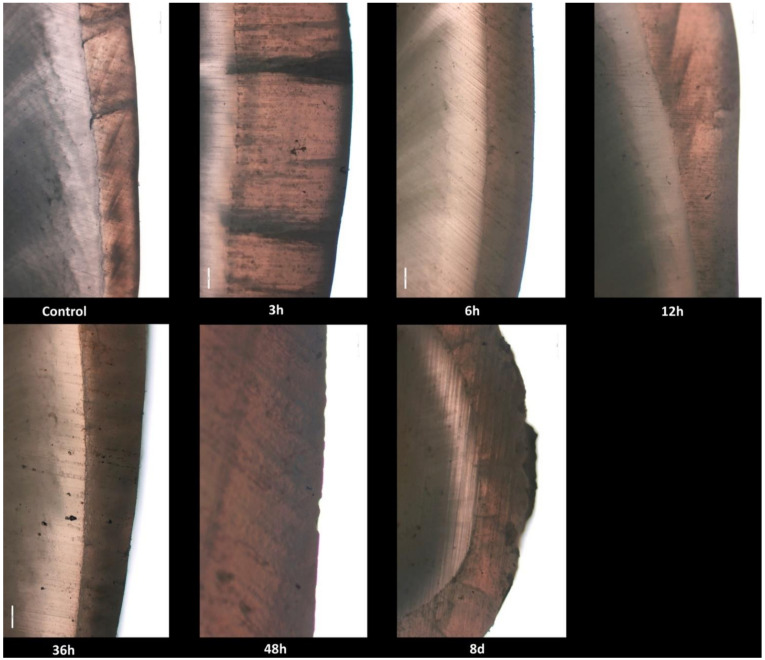
Photomicrographs of enamel and dentin surface morphology in the control group and radioiodine exposed groups at 3, 6, 12, 36, 48 h and 8 days.

No histological changes in the enamel structure and morphology were identified in the control group. Normal histological features of tooth enamel were also observed at 3, 6, 12 and 36 h after exposure, respectively. At 48 h after exposure, the enamel surface showed multifocal and irregular areas with distinctive roughness. At 8 days after exposure, multiple, very deep and delimited cavities with elevated margins were revealed in the enamel structure (Figure 1).

### 3.2. SEM Findings

**Figure 2:**
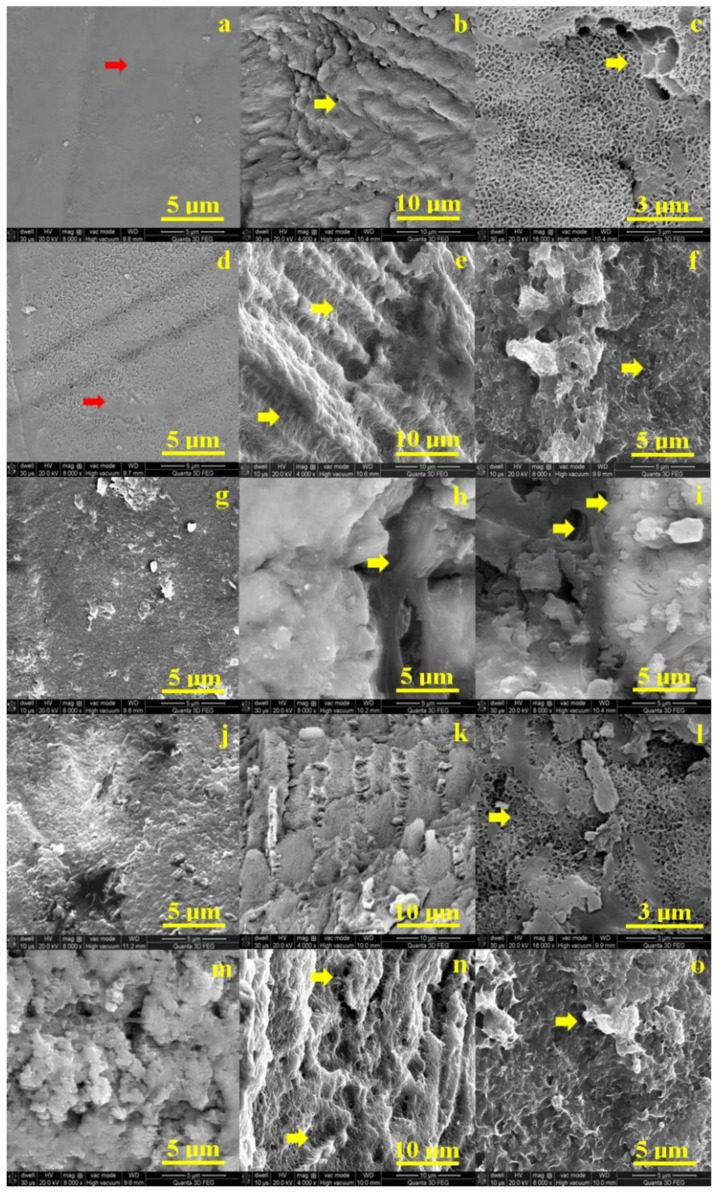
Scanning electron micrograph of: enamel—no exposure (**a**), exposed for 6 h (**d**), 12 h (**g**), 48 h (**j**) and 8 days (**m**); interface—no exposure (**b**), exposed for 6 h (**e**), 12 h (**h**), 48 h (**k**) and 8 days (**n**); and dentine slab—no exposure (**c**), exposed for 6 h (**f**), 12 h (**i**), 48 h (**l**) and 8 days (**o**).

Figure 2a shows the SEM aspect of the untreated, healthy enamel. It features a uniform surface with a very compact structure of prisms. The structural compactness is assured by small HAP nano-particles (very well bonded each to another by small protein units inside of the enamel rods (prisms). They are not visible due to the high quality of the enamel surface. Slightly alterations of the enamel surface occur after 6 h of exposure to the I-131 treatment. The protein binder into the surface is weakened by the radiation and nano HAP crystal loss occurs. It leads to small pitches to appear on the surface (Figure 2d). These superficial alterations have rounded shape and diameters vary from 80–150 nm.

Significant morphology changes involves after 12 h of treatment (Figure 2g). The HAP crystal loss is extended to the microstructure level which affects the surface cohesion. The dislocated HAP debris is observed as bright spots over the surface. Their general aspect is rounded having diameters in a wide range from 150 nm to over 1 mm. The most representative ones are the 3 micro-sized particles in the upper right side of the SEM image. We noticed some submicron HAP debris agglomeration situated on the middle top of the SEM image in Figure 2g. After 48 h the enamel surface is profoundly altered by large micro-size depressions having a diameter of about 5 μm. Fact is correlated with significant loss of mineral material within the enamel prisms (Figure 2j).

The initial state of dentine is observed in Figure 2c. It appears a compact network of collagen fibbers strongly mineralized with HAP nano-crystals. This structure is the intra-tubular dentine, a significant dentine tubule is observed in the right upper corner of the SEM image in Figure 2c. The dentine surface after 6 h of treatment is more rugged than the initial state but there no appears sings of mineral loss or structural alterations, Figure 2f.

The structural alterations occur in dentine after 12 h of treatment (Figure 2i). Several depressions alter the observed surface especially in the center of the observation field and on the left side of the image. It seems to appear some boulder formations on the surface ranging from 1 to 3.5 μm. The degradation continues by increasing of the depressions featuring mineral loss tendency after 48 h of treatment. The mineral loss makes more visible the collagen fibers network which surrounds the boulder formations which are more visible. The profound decay of dentin is observed after 8 days (Figure 2o). The mineral loss is profound in the surface which embosses the collagen fibers network; the most representative boulder formations are indicated by a yellow arrow.

**Figure 3:**
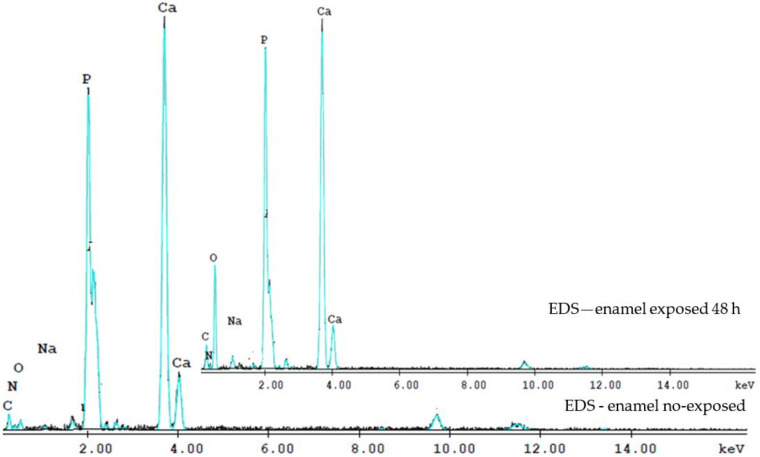
The EDS spectra evidence the Ca and P contents of the enamel no-exposed and exposed 48 h to the laser irradiation. The Ca/P ratio at 48 h was lower than that of no-exposed enamel.

The complexity of the phenomena involved in the spectroscopic techniques triggered by the electron beams, makes precise quantitative analysis extremely difficult. The obtained EDS spectra show the bands of elements present in the enamel matrix, and other traces of elements (Figure 3). The EDS analysis measured the relative intensities of Ca and P in the total element contained. The intensities were normalized areas, in weight of the total content of the elements and the atomic percentage (percent), due to the different regions of interest chosen.

Thus, we used analyzes to determine the Ca/P ratio. The Ca/P ratio at 48 h was lower than that of the intact enamel, suggesting the loss of calcium-deficient apatite. On the enamel surface, Ca and P content in the enamel no exposed sample is higher than that after exposed to irradiation at 48 h. The Ca/P ratio in the enamel control sample of 1.69 ± 0.11, close to the Ca/P ratio of hydroxyapatite [Ca_10_(PO_4_)_6_(OH)_2_] of 1.65, while the Ca/P ratio for irradiated enamel at 48 h was 1.43 ± 0.09.

The oxygen content of irradiated teeth also increased at 48 h. Radiation interacts with organics and water and induces free radicals and hydrogen peroxide in dental hard tissue, hence the high oxygen content in irradiated teeth 48 h. An irregular increase and decrease of Na and Mg elements was observed, and of the Ca/P weight ratio in irradiated teeth and at 6, 8, 12, 48 h and 8 days.

### 3.3. AFM Findings

**Figure 4:**
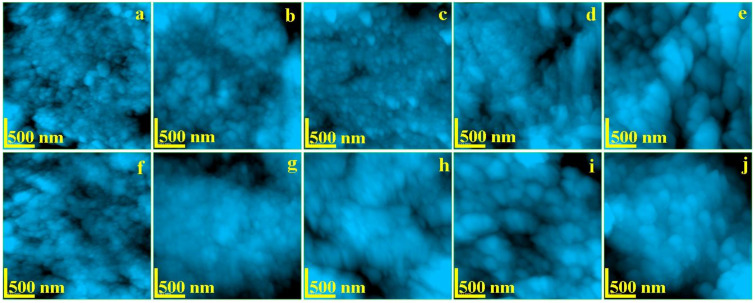
AFM topographic images of: Enamel—(**a**) no exposure, (**b**) exposed 6 h, (**c**) exposed 12 h, (**d**) exposed 48 h, and (**e**) exposed 8 days; Dentine—(**f**) no exposure, (**g**) exposed 6 h, (**h**) exposed 12 h, (**i**) exposed 48 h, and (**j**) exposed 8 days. Scanned area 2.5 × 2.5 μm.

The enamel and dentine nanostructure was investigated by AFM at a scanning area of 2.5 × 2.5 μm to assure an optimal view of the ultra-structural features. The resulted images are presented in Figure 3. The topography of healthy untreated enamel (Figure 4a), is formed by a very compact structure of HAP nano-crystals having diameter of about 40 nm which are bonded together by protein units. This is the typical nano-structure which assures the good quality observed by SEM in Figure 2a.

The topography of healthy untreated dentine is presented in Figure 4f. The HAP mineralized collagen fiber network forms a compact structure. The diameter of HAP nano-particles is about of 40 nm. These are strongly bonded by the collagen assuring a very tenacious material.

The radiation affects mainly the organic binder of the HAP nano-particles as previously observed. It was observed that the dentine is slowly affected by radiation after 6 h of exposure (Figure 4g). The collagen tends to lack the binding effect still maintaining the HAP diameter to about 40 nm. This tendency is more accelerated after 12 h in Figure 4h, where the HAP nano-particle diameter increases up to 45 nm. Large amount of collagen binding failure is observed after 48 h of treatment (Figure 4i). It leads to mineral loss materialized by submicron depressions occurrence combined with a HAP diameter increasing up to 65 nm. Fact is in good agreement with the micro structural changes observed by SEM in Figure 2l. The deeper decay of the dentine nanostructure occurs after 8 h of treatment within a diameter of the HAP nano-particles around 85 nm. The dentine alterations caused by the radiation lead to a progressive increasing of the roughness but in a mild manner compared to the enamel (Table 1). Fact may be explained by dentine position inside of the tooth which allows a small retardation of the irradiation effect.

## 4. Discussion

The human enamel proves to be affected by the activities of I-131 radioiodine, used in the ablation therapy of DTC. Therefore, SEM imaging was employed to investigate the morphology changes at several radiation exposures. The enamel destruction progresses along with the treatment time increasing. The alterations are extended into the enamel depth and the dislocated HAP debris is overwhelming. It consist in micro sized boulders having diameters in a range of about 1–2 μm.

The enamel-dentine interface was also observed by SEM imaging. The initial sample evidences a very strong cohesion between enamel and dentine (Figure 2b). Small gaps occur at the interface after 6 h of treatment. Their shape is rounded, having diameters between 2 to 5 μm and are positioned along the interface line. The gap evolves in time. It is a very well developed valley after 12 h of treatment (it is indicated by yellow arrow in Figure 2h), having a width of about 2 μm. The interface after 48 h becomes fuzzy due to the mineral loss debris (Figure 2k). Finally, the interface microstructure resulted after 8 days of treatment is deeply altered (Figure 2n). It is difficult to figure out the border line, but some hints about it are indicated by yellow arrows.

The morphology aspects observed by SEM images allows us to observe that the enamel tends to decay faster than the dentine. This fact is due mainly to its position on the exterior side of the tooth and because of small protein binder amount within it. The position of dentin inside of the tooth correlated with the significant amount of collagen present there explains the slower decay rate. The alterations in both enamel and dentin appear between 12 and 48 h and after 8 days of treatment are extended in depth.

The AFM imaging shows that I-131 treatment affects the protein bond between HAP nano-crystals causing loss of cohesion. This fact leads to significant increasing of nano-particles diameter: 60 nm after 6 h of treatment and finally 85 nm after 8 days. The cohesion loss facilitates mineral loss observed by SEM which leads to a significant increasing of the surface roughness. Nano depressions are observed after 12 h of treatment with an diameter of about 500 nm (Figure 4c). These depressions increase in diameter after 48 h of treatment (e.g., 800 nm). Finally, the nanostructure is deeply affected after 8 days of treatment.

The mechanism of I-131 therapy on tooth structure remains unspecific, given the small number of research available in the specialty literature. Information regarding the influence of radiotherapy on the bone structure are more commonly found. General effects of radiotherapy on bone density are widely known, but specific effects of radioiodine therapy on bone structure were insufficient investigated. Research showed that modifications in the architecture of bones can occur beginning with the second week after radiation, underlining their role in manifestations of bone brittleness and fractures [20,21,22]. In vivo clinical studies showed that the elevated activity of osteoclasts and accelerated bone destruction represent consequences of massive quantities of radiations [23]. Clinical exposure of necrotic bone in the oral cavity has also been observed in patients undergoing radiation therapies. Histopathological research showed that high activities of collagen type I and fibrosis are present in osteoradionecrosis of the jaws [24].

The influence of radioiodine therapy on the periodontal structures depends on the health condition of the oral soft tissues, prior to the initiation of radiation therapy, but also on the dose of radioiodine prescribed for each specific case [17]. Periodontal defects or mixed endodontic-periodontal disorders can occur after radioiodine therapy [17]. Gingival inflammation or hemorrhages are also cited among the undesired side effects of I-131 treatment. High-dose radioiodine treatment can impair the long-term oral health, depending on the cumulative radioiodine activity and individual salivary gland radioiodine uptake [25]. Moreover, I-131 therapy influences the values of prostaglandins and contributes to the occurrence of gingival and periodontal inflammation [26]. In order to prevent periodontal structure loss and the appearance of incipient teeth decays following I-131 therapy, Amdur and collaborators [27] are recommending teeth prophylaxis (scaling, root planning, every 3 months), respect of oral hygiene on a daily basis, the use of toothpaste with high fluoride concentration (at least two brushing/day) and custom trays with fluoride gels.

Given the lack of studies available, the present article represents an original initiative, supporting the future investigations regarding the side effects of radioiodine treatment on human enamel and dentin structure and even more, on bone metabolism behavior. However, limits of the conducted study must be taken into consideration. The relative enclosed number of teeth that have been used, the in-vitro character of the investigations and the deficiency of other studies available for comparison serve as starting point for investigations for the vast mechanisms of radioiodine therapy in relation to the human oral cavity.

## 5. Conclusions

After radioiodine-131 exposure, a slight alteration of the enamel had been seen after 6 h. After 12 h, significant morphology changes with hydroxyapatite crystal loss are detected in enamel, which determines alteration of surface cohesion. Also, structural changes can be seen in dentine surface with appearance of boulder formations. This degradation continues by increasing the depressions featuring mineral loss tendency after 48 h of treatment. After 8 days, the interface microstructure is deeply altered with increased depressions in diameter.

## Figures and Tables

**Table 1:** Parameters measured with AFM on enamel and dentine samples.

Parameter	Treatment Time, Hours				
	0	6	12	48	192
	Enamel				
Ra (nm)	19.2	26.5	28.3	40.0	73.4
Rq (nm)	26.6	36.3	39.4	50.2	52.4
Diameter (nm)	40	60	75	80	85
	Dentine				
Ra (nm)	19.4	29.7	31.8	35.3	40.2
Rq (nm)	24.0	36.1	39.6	40.6	50.4
Diameter (nm)	40	40	45	65	70

## Data Availability

The data presented in this study are available on request from the corresponding authors.
